# SERPINA3 predicts long-term neurological outcomes and mortality in patients with intracerebral hemorrhage

**DOI:** 10.1038/s41419-025-07551-x

**Published:** 2025-03-29

**Authors:** Pei Zheng, Zhihui Qi, Bin Gao, Yang Yao, Jingshan Chen, Hengri Cong, Yue Huang, Fu-Dong Shi

**Affiliations:** 1 Department of Neurology, China National Clinical Research Center of Neurological Diseases, Beijing Tiantan Hospital, Capital Medical University, Beijing, China; 2 Department of Neurology, Tianjin Medical University General Hospital, Tianjin, China; 3 Tiantan Brain Bank, Beijing Tiantan Hospital, Capital Medical University, Beijing, China

**Keywords:** Predictive markers, Stroke, Brain injuries

## Abstract

Intracerebral hemorrhage (ICH) is a severe stroke subtype with high mortality and disability rates, and long-term outcomes among survivors remain unpredictable due to the lack of reliable biomarkers. In this study, spatial transcriptomics was used to analyze molecular profiles in autopsy brain tissues from chronic ICH patients, revealing distinct transcriptomic features in the thalamus and cortex, with common inflammatory characteristics such as gliosis, apoptosis, and immune activation. Serine proteinase inhibitor NA3 (SERPINA3) was significantly upregulated in both regions and co-expressed with astrocytes in the thalamus. Pathological studies in postmortem human tissues and mouse models confirmed elevated SERPINA3 expression, with murine Serpina3n showing a similar pattern in mice. Plasma analysis of 250 ICH patients and 250 healthy controls revealed significantly higher SERPINA3 levels in ICH patients, correlating with hemorrhage severity, National Institutes of Health Stroke Scale (NIHSS), and Glasgow Coma Scale (GCS) scores, and long-term functional outcomes. Higher SERPINA3 levels within 72 hours of hemorrhage onset were independently associated with worse functional recovery (mRS ≥ 3) and increased all-cause mortality at 6 and 12 months. Additionally, SERPINA3 levels at 7 days post-ictus correlated with white matter hyperintensities and poor cognitive performance at 6 months. These findings highlight SERPINA3 as a potential prognostic biomarker for ICH, warranting further investigation into its role in long-term neurological dysfunction and validation in larger prospective cohorts.

## Introduction

Intracerebral hemorrhage (ICH) is a devastating neurological condition associated with significant morbidity and mortality [1, 2]. Although acute symptoms are directly caused by the structural damage from the hematoma, long-term neurological deficits are not strongly associated with the acute characteristics of ICH, suggesting that different mechanisms may underlie long-term neural damage [3]. Secondary brain injury following cerebral hemorrhage is not caused by a single factor, but rather by a series of amplified reactions involving multiple pathological factors. These include excitotoxicity, apoptosis, amyloid deposition, disruption of the blood-brain barrier, and neuroinflammation [4, 5]. These, along with other unidentified pathogenic events, may determine the long-term functional deficits, such as verticality misperception, reduced verbal fluency performance, and poor motor, cognitive, and emotional outcomes after ICH. Early prediction of outcomes in patients with ICH is crucial for readiness of patients and their caregivers. Biomarkers could help individualize care by stratifying the risk of secondary brain injury, optimizing stroke management, improving rehabilitation, and enhancing overall stroke outcomes.

For this need, neuroimaging techniques, including MRI and CT scans, can now thoroughly describe the characteristics of the bleeding lesion. Established imaging markers, such as perihematomal edema and white matter integrity, can partially assist in predicting neurological outcomes in ICH patients [6–9]. However, the resolution of neuroimaging abnormality is insufficient to detect changes at the cellular and molecular levels of the brain after ICH, limiting its ability to provide more accurate and reliable prognostic information. Biomarkers, which can objectively evaluate normal biological processes, pathogenic mechanisms, and responses to interventions, are emerging as a promising new tool for predicting stroke prognosis. For instance, the plasma neurofilament light chain is a sensitive marker for monitoring axonal injury post-ICH and can predict long-term functional outcomes and survival [10]. Additionally, C-reactive protein (CRP), white blood cell (WBC) count, and changes in leukocyte count over the first 72 hours after admission also show prognostic value [11].

Previous studies have shown that neuroinflammation can persist throughout different stages of cerebral hemorrhage. In the acute phase, injury-related molecular patterns released from damaged neural structures rapidly activate the immune system, leading to infiltration of peripheral immune cells into the brain and exacerbating local neuroinflammation. The evolution of neuroinflammation during the chronic stage after ICH is less clear. Some evidence suggests that inflammation may persist and spread long after ICH, potentially contributing to long-term outcomes [12–14]. If confirmed, the identification of new inflammatory biomarkers could enhance current panels and improve prognosis prediction. The current study aims to test this hypothesis.

## Materials and Methods

### Human brain tissue and blood samples

#### Brain tissue

Human brain sections were acquired from Beijing Tiantan Hospital (Beijing, China), as previously described [15]. Of the 6 brains studied, 3 were from deceased ICH patients, and 3 were from deceased individuals with no history of neurological or neuropsychiatric diseases. The ICH patients and control subjects had similar mean ages at death (ICH: 63.4 ± 2.2 years of age; control: 59.8 ± 2.3 years of age; mean ± SEM; *p* > 0.05; unpaired t test). Brain tissues were collected within 4 hours post-mortem.

#### Subjects and samples

This study analyzed data from the Chinese Cerebral Hemorrhage Mechanisms and Intervention study cohort (ChiCTR1900020872). Human blood samples were obtained from 250 healthy controls and 250 patients with ICH. The inclusion criteria for the study were patients above 18 years old, presenting with a first-episode ICH within or exactly at 24 hours. Diagnoses of ICH followed the Guidelines for Diagnosis and Treatment of Cerebral Hemorrhage [16]. Patients with primary subarachnoid hemorrhage, cerebral infarction hemorrhage transformation, hemorrhage post-thrombolysis, traumatic cerebral hemorrhage, subdural/extradural hematoma, and other diseases (neurodegenerative diseases, tumors, cognitive impairment, dementia) were excluded. All study participants had no prior history of neuropsychiatric disease, stroke, dementia, or underlying diseases at the time of blood collection. Plasma samples from participants were processed according to protocols approved by the local ethics committee (No: S485). Written informed consent was obtained from each patient or their legal surrogate.

At recruitment, attending physicians collected detailed clinical histories from the prospective participants, with additional relevant information obtained through a review of past medical records. Patients were assessed with the Glasgow Coma Scale (GCS) and National Institutes of Health Stroke Scale (NIHSS) at admission and the time of blood collection. A diagnosis of diabetes was recorded if patients were receiving pharmacological anti-diabetic treatment, and hypertension was considered if patients were on antihypertensive medication prior to admission. Smoking and alcohol abuse status were recorded from the medical charts. Computed tomography (CT) scans were evaluated by a skilled neuroradiologist, and ICH volume was quantified using the ABC/2 method. Routine follow-ups were conducted to evaluate functional outcomes with the mRS at 1, 3, 6, and 12 months, via either telephone or face-to-face interviews, until June 2021 or death. Twenty-six patients underwent further cognitive evaluation by Montreal cognitive assessment (MoCA) and brain structural assessment via multi-modal magnetic resonance imaging (MRI) at 6 months post-ICH.

### Human: GeoMx spatial transcriptomics

Spatial transcriptomics was performed using the NanoString GeoMx® Digital Spatial Profiler. Tissue sections were cut at a thickness of 6 μm and mounted on plus-charged slides (Leica BOND Plus slides). All subsequent steps were carried out under RNase-free conditions using DEPC-treated water. The slides were then deparaffinized through three sequential 5-minute washes in xylenes, followed by two 5-minutes washes in 100% ethanol, 1 wash in 95% ethanol, and 1 wash in 1x PBS. Target retrieval was performed using a target retrieval reagent (10x Invitrogen 00-4956-58 EDTA pH 9.0) diluted to 1x in the BioGenex EZ-Retriever System for 10 minutes at 95 °C. After washing in 1× PBS, the slides were incubated overnight at 37 °C with RNA hybridization probes (NanoString, GMX-RNA-NGS-HuWTA-4) diluted in Buffer R (provided in the GeoMx RNA Slide Prep formalin-fixed paraffin-embedded (FFPE)- PCLN kit, GMX-PREP-RNA-FFPE-PCLN-12). The following day, slides were stained with antibodies Iba1 (CST, 48934), GFAP (Novus, NBP2-33184AF532), NeuN (Abcam, ab190565), and Syto13 (Nanostring, GMX-RNA-MORPH-HST-12) for 1 hour at room temperature, then loaded into the GeoMx DSP instrument for scanning and selecting the regions of interest (ROI). A total of 46 ROIs were selected, including 22 in the cortex and 24 in the thalamus. Immunofluorescence for DAPI was used to identify morphological markers. Iba1, NeuN and GFAP were used for segmentation. ROIs selected for subsequent gene expression analysis were marked with circles, and each ROI was segmented into three areas of-illumination (AOI): Iba1 positive for microglia, NeuN-positive for neurons, and GFAP-positive for astrocytes. Probe identities in each segment were captured via UV illumination and transferred to a 96-well plate. The PCR system was constructed following the DSP standard process, and the library was generated.

### Digital spatial profiling analysis

Raw FASTQ files were converted into digital count files using the GeoMx NGS Pipeline software (v2.0). Segment and Probe quality control (QC) was performed to remove undesirable AOI/ROI and outlier probes. The quality control (QC) section includes 3 parameters: Aligned reads and Stitched read > 80%, Sequencing saturation > 50%. The limit of detection (LOQ) was established for each segment, and gene expressions below the limit of quantitation were excluded. The LOQ was determined by multiplying the geomean of the negative probes by their geometric standard deviations. A total of 8,022 genes were detected (with the filtering criterion being that the gene’s expression exceeded LOD in at least 4 AOIs). Data were normalized using Quantile 3 normalization after completing the quality control steps. For differential gene expression analysis, edgeR was employed based on the DSP-WTA technology platform [17]. We compared the differences between AOI groups (ICH and controls). Genes with a false Discovery Rate (FDR) < 0.05 and log2 (Fold Change) > 1, were considered differentially expressed genes (DEGs). The identified DEGs were analyzed using the Toppgene Suite mainly for gene ontology annotation. Upregulated and downregulated genes were further enriched by GO and KEGG by ORA method. Gene signature analysis was performed using the Hallmark gene collection from the MSigDB (Molecular Signatures Database) database. Statistical tests were applied to test hypotheses in selected groups. The Wilcoxon rank sum test was used to compare two groups of independent AOI/ROI, while the Kruskal-Wallis method was used to analyze the rank sum across multiple independent AOI/ROI groups.

### Animals

Male and female C57BL/6 mice, aged 2-3 months, were purchased from SPF Biotechnology Co., Ltd. (Beijing, China). The mice were housed in facilities under standardized light-dark cycle conditions with access to food and water ad libitum. All animal experiments were approved by Animal Care and Use Committees of Beijing Tiantan Hospital, Capital Medical University (201902026). All procedures were performed in accordance with the National Institutes of Health Guide for the Care and Use of Laboratory Animals and were designed and performed according to the Animal Research: Reporting In Vivo Experiments guidelines (www.nc3rs.org.uk/arrive-guidelines).

### Induction of murine ICH model

ICH was induced in mice by injection of autologous blood or collagenase as previously described [15, 18]. Mice were anesthetized with a ketamine/xylazine mixture via intraperitoneal injection and positioned prone in a stereotactic head frame. A 1-mm-diameter hole was drilled on the right side of the skull (2.3 mm lateral to midline, 0.5 mm anterior to bregma). In the autologous blood model, 30 μl of non-heparinized blood was withdrawn from the angular vein and infused using an infusion pump. Initially, 5 μl was injected at a rate of 1 μl/min at a depth of 3.0 mm, followed by the remaining 25 μl at the same rate at a depth of 3.7 mm. The needle remained in place for 15 minutes to prevent back flow before being gently withdrawn. In the collagenase model, 0.0375 U of bacterial collagenase (type IV, Sigma-Aldrich, St. Louis, MO) in 0.5 μl of saline was administered at the same coordinates at a rate of 0.5 μl/min. Sham-operated groups underwent a similar surgical procedure with an equal volume of sterile saline injection. The cranial burr hole was sealed with bone wax, and the incision was sutured. Body temperature was maintained at 37.0 ± 0.5 °C throughout the procedures. The overall mortality rate of mice subjected to ICH was approximately 4.8%.

### Western blot

Thirty-five days after ICH, the ipsilesional brains of mice were dissected, and proteins were extracted using Minute™ Total Protein Extraction Kit for Animal Cultured Cells/Tissues (Invent). Proteins were analyzed using 10% SDS-PAGE and transferred onto a PVDF membrane (Millipore). The membrane was blocked with Rapid protein free blocking buffer (Beyotime) for 15 minutes at room temperature, followed by incubation with primary antibodies: anti-mouse Serpin A3N (R&D Systems, AF4709, 1:1000) and β-actin (Abcam, ab8226, 1:5000) overnight at 4 °C. After three washes, the membrane was incubated with horseradish peroxidase-conjugated rat anti-mouse (Invitrogen, 04-6020, 1:5000) and goat anti-rabbit secondary antibodies (Invitrogen, 65-6120, 1:5000) for 1 hour at room temperature. The signals of the detected proteins were visualized with an ECL detection system (Bio-Rad), and the intensity of each band was quantified using ImageJ software (National Institutes of Health).

### Immunofluorescent staining

On the 35th day after surgery, mice were anesthetized and perfused with pre-chilled phosphate-buffered saline (PBS), followed by 4% paraformaldehyde (PFA). The brains were then fixed in 4% PFA and sequentially dehydrated in 15% and 30% sucrose solutions. The dehydrated brains were embedded in optimal cutting temperature (OCT) compound (Sakura) and sectioned into 5-μm-thick continuous coronal slices. The sections were permeabilized and blocked with 5% donkey serum and 0.3% Triton X-100 in PBS for 2 hours at room temperature. This was followed by incubation with primary antibodies overnight at 4 °C. After three times washes in PBS, the slices were incubated with appropriate fluorochrome-conjugated secondary antibodies at room temperature for 2 hours. The slices were washed again as described above and then incubated with Fluoroshield Mounting Medium with 4′,6-diamidino-2-phenylindole (DAPI) (Abcam). The following primary antibodies were used: anti-mouse Iba1(Abcam, ab178847; 1:200), anti- mouse GFAP (Abcam, ab7260;1:500), anti- mouse NeuN (Abcam, ab177487; 1:200), anti-mouse Serpin A3N (R&D Systems, AF4709, 1:100), donkey anti-rabbit AF546 (A11056, Invitrogen;1:1000), donkey anti-goat AF488 (A11055, Invitrogen;1:1000). Images were captured using a fluorescence imaging multifunctional detection system (BioTek Citation 5) and quantified by Image J (U.S. National Institutes of Health).

### Blood sampling and Serpin A3 measurement

Peripheral blood samples were obtained using ethylenediaminetetraacetic acid (EDTA) tubes within 72 hours of ICH onset. Blood samples from 41 patients were collected randomly on days 7 and 14 post-ICH, with clinical characteristics presented in Table 1. The samples were centrifuged at 3000 rpm for 5 minutes, and the separated plasma was preserved at -80 °C. Plasma Serpin A3 levels were measured using commercial human Serpin A3 enzyme-linked immunosorbent assay (ELISA) kits (KALANG, KL-0570H), with a limit of detection (LoD) of 0.5 ng/ml. Each sample was measured twice using the same kit. The ELISA plate was read by Varioskan Flash 4.00.53 (Thermo Fisher Scientific).

**Table 1:** Association of SERPINA3 concentrations with ICH volume at blood collection.

	Adjusting for the time from hemorrhage to blood collection			Adjusting for the time from hemorrhage to blood collection, age at blood collection, sex, current smoking, hypertension, cerebrovascular disease, and diabetes	
Variable types	*N*	β (95%CI)	*P*-value	β (95%CI)	*P*-value
Overall	250	3.38 (2.45, 4.31)	<0.001	3.2 (2.26, 4.14)	<0.001
**Hemorrhage location**					
No	220	3.67 (2.65, 4.70)	<0.001	3.46 (2.42, 4.49)	0.001
Yes	30	0.51 (−0.49, 1.50)	0.306	1.05 (−0.09, 2.18)	0.069
**Ventricular extension**					
No	182	2.88 (1.82, 3.95)	<0.001	2.6 (1.52, 3.68)	<0.001
Yes	68	4.19 (2.32, 6.05)	<0.001	4.04 (2.10, 5.98)	<0.001
**Surgery**					
No	140	0.71 (0.27, 1.16)	0.002	0.62 (0.16, 1.08)	<0.001
Yes	110	2.1 (0.58, 3.62)	0.007	2.21 (0.71, 3.70)	0.004

*β* regression coefficient, *CI* confidence interval. The β values, 95%CIs, and p-values were obtained from the linear regression models. β values were interpreted as the change in mean ABC/2 scores for each fold of SERPINA3 concentration. The bleeding volume of intravascular hemolysis (IVH) was calculated using a 3D slicer.

### MRI protocols and imaging analysis

MRI scanning was performed on 26 patients six months after ICH. All MRI data were acquired using a 3.0-Tesla GE MR scanner (SIGNA Pioneer, General Electric, Milwaukee, WI, USA) at the Third People’s Hospital of Datong. The brain MRI sequences included: axial 3-dimensional (3D) T1-weighted images (TR/TE = 8.3/3.2 ms, flip angle = 12°, FOV = 240 mm × 240 mm, slice thickness = 1 mm, gap = 0, slice number = 156), T2-weighted fluid-attenuated inversion recovery (FLAIR) (TR/TE/TI = 8000/120/2100 ms, FOV = 240 mm×240 mm, slice thickness = 5.0 mm, gap = 1 mm, slice number = 23) and DTI (TR/TE = 8000/77 ms; FOV = 240 mm×240 mm; slice thickness=5 mm with no gap; 32 encoding diffusion directions with one values of b [*b* = 1000] for each direction and 10 non-diffusion-weighted images [b = 0 s/ mm2], slice Number=30).

White matter lesions were marked by a professional neuroradiologist on T2- FLAIR images using a 3D-Slicer (https://www.slicer.org/). DTI data was preprocessed using the FMRIB’s Diffusion Toolbox (FDT, http://www.fmrib.ox.ac.uk/fsl, FSL 4.0). First, eddy current distortions and motion artifacts were emended using the affine alignment of each diffusion-weighted image to the image of *b* = 0 s/mm2. Next, non-brain tissue was removed from the average *b* = 0 image using FMRIB’s Brain Extraction Toolbox (BET), and a brain mask was applied to the remaining diffusion-weighted images. The fractional anisotropy (FA) map was derived by estimating the diffusion tensor for each voxel using the DTIFIT function via linear regression. The FA map was then registered to MNI space using FLIRT linear registration. For further analysis, normalized, modulated, and smoothed white matter density maps were created as masks to extract FA values from patients. Gray matter volume was evaluated using voxel-based morphometry (VBM) analysis, performed with the CAT12 Toolbox (http://www.neuro.uni-jena.de/cat) implemented in the statistical parameter mapping software (SPM12, http://www.fl.ion.ucl.ac.uk/spm). T1 images were normalized to template space, followed by segmentation of gray matter, white matter, and cerebrospinal fluid.

### Statistical Methods

For all analyses, a significance level of 0.05 was used, and statistical analyses were performed using IBM SPSS version 19.0 (IBM Corp., Armonk, N.Y., USA) and Prism 8.0.2 software (GraphPad). Experimental groups, data collection, and data analysis were blinded by using different investigators or masking sample labels. For animal experiments, the sample size was estimated by experience and mice were randomly allocated to their respective experimental groups. Sample exclusion was done because of mouse death after surgery. For plasma experiments, the priori power analysis calculates the required sample size based on the desired power, effect size, and significance level. The normality of data distribution was assessed using the Shapiro–Wilk test. Continuous variables are described as means ± SEM or median (interquartile range), while categorical variables are shown as numbers and percentages. After adjusting for age, then we used Mann–Whitney U test to compare plasma SERPINA3 levels between ICH patients and healthy controls. SERPINA3 levels were assessed using a base-2 logarithmic transformation to account for their skewed distribution. In patients with ICH (overall and stratified according to hemorrhage location, ventricular extension, surgery), the relationships between SERPINA3 with hemorrhage volume at admission (ABC/2), NIHSS, and GCS, and outcomes at 1, 3, 6, 12 months (mRS and survival) were examined using appropriate regression models. Linear regression models were used to assess the associations between SERPINA3 and ABC/2, NIHSS, and GCS, with these variables examined on the square root scale due to their skewed distributions. β coefficients and 95% confidence intervals (CIs) were estimated and interpreted as changes in the mean ABC/2, NIHSS, and GCS scores (on the square root scale) for each fold of SERPINA3 concentration. Associations between SERPINA3 and mRS scores were analyzed using binary logistic regression models, with mRS dichotomized as < 3 or ≥ 3. Odds ratios (ORs) and 95% CIs were estimated. Additionally, Cox proportional hazards regression models were utilized to evaluate the link between SERPINA3 concentrations and survival. The baseline time point for survival analysis was defined as the date of blood collection, and patients were censored at the date of the last follow-up. Hazard ratios (HRs) and 95% CIs were estimated. All HRs and ORs corresponded to a doubling in the SERPINA3 concentration. All regression models (Cox, linear, logistic) were initially adjusted for the time from ICH onset to blood collection. Further adjustments were then made for variables such as age, sex, smoking status, hypertension, history of cerebrovascular disease, and diabetes. We further evaluated the ability of SERPINA3 to independently predict 6- and 12-month mRS scores and 12-month survival using Cox regression models. When evaluating survival at 12 months, the area under the receiver operating characteristic (ROC) curve (AUC) was computed. The optimal cut-off point was identified as the SERPINA3 value that provided the highest correct classification, and the positive/negative likelihood ratio for that point was determined. Kaplan–Meier survival curves were analyzed using the log-rank test for predictors above and below the cut-off point. For 6-month and 12-month mRS (categorized as <3 or ≥3), the AUC was estimated both with and without SERPINA3 in the multivariable model. Associations between SERPINA3 concentrations, image markers, and cognitive status were assessed using Spearman’s rank correlation test.

## Results

### Spatial transcriptomics reveals upregulation of SERPINA3 in brain autopsy tissues from chronic stage of ICH patient

**Fig. 1:**
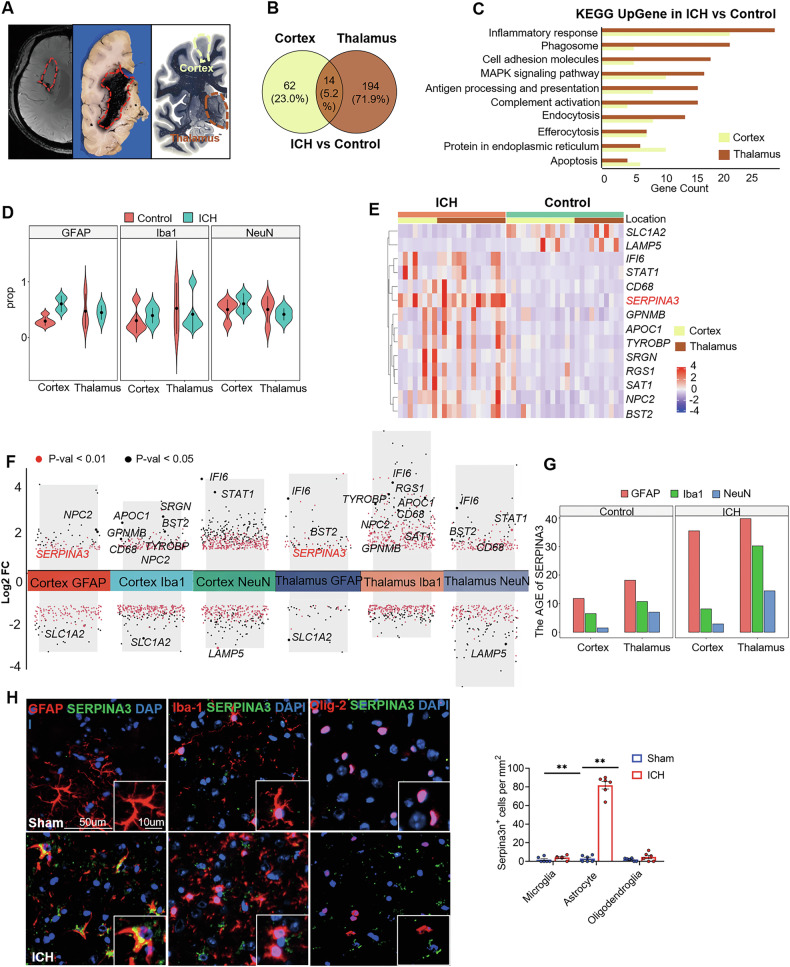
Spatial transcriptomics reveals upregulation of SERPINA3 in thalamus and cortex from chronic stage of ICH patient. **A** Left: the representative T2/Magnetic-sensitive weighted MR image; Middle: Brain autopsy tissue from a patient in the chronic stage of ICH; Right: Location diagram denotes the cortex and thalamus regions. Thalamus and cortex tissues were collected from patients with right basal ganglia hemorrhage who died 6 years after onset (*n* = 3). Subjects without a history of neurological disease were used as controls (*n* = 3). **B** Venn diagrams showing common and unique differentially expressed genes between the thalamus and cortex. **C** Kyoto Encyclopedia of Genes and Genomes (KEGG) analysis of DEGs in the thalamus and cortex. **D** The violin diagram shows changes in the number of astrocytes, microglia and neurons between the thalamus and cortex in ICH patients compared to controls. **E** Heatmap displaying the relative average expression of common DEGs between the thalamus and cortex in ICH patients compared to controls. (F) Differential gene expression in astrocytes, microglia, and neurons of the thalamus and cortex in ICH patients compared to controls. Common DEGs between the thalamus and cortex are shown. The Y-axis represents average log_2_FC. Red indicates an adjusted *P* value < 0.01. **G** The average expression of SERPINA3 in the GFAP, Iba-1 and NeuN segmentation from thalamus and cortex between controls and ICH patients. **H** Immunostaining and quantification of SERPINA3 expression in microglia (Iba1^+^ cells), astrocytes (GFAP^+^ cells), and oligodendrocyte (Olig-2^+^ cells) in the thalamus from chronic stage of ICH patients and controls. *n* = 6 per group. Scale bar, 50 μm (Insert: 10 μm). Unpaired two-tailed t test. Data are presented as means ± SEM, **P < 0.01.

We performed spatial transcriptomics using the GeoMx Digital Spatial Profiler (DSP) to identify molecules whose expression and translation levels could better explain the chronic phase of ICH compared to controls, with the aim of identifying potential biomarkers. Previous studies have highlighted the crucial roles of the cortex and thalamus in cognitive processes. The thalamus-driven functional networks in the frontal cortex are particularly important for decision-making [19]. Damage to either the cortex or thalamus can lead to varying degrees of cognitive dysfunction. We isolated cortex and thalamus tissues from postmortem brains of patients with a 6-7 years history of ICH (n = 3) and compared them to age- and sex-matched controls with no history of neurological disease (*n* = 3) (Fig. 1A**)**. In contrast to the three healthy controls, the cortex and thalamus of ICH patients exhibited distinct gene expression patterns. A total of 76 differentially expressed genes (53 upregulated and 23 downregulated, fold change > 1, *p* < 0.05) were identified in the cortex, and 208 differentially expressed genes (162 upregulated and 46 downregulated, fold change > 1, *p* < 0.05) were identified in the thalamus when comparing ICH patients to controls (Supplementary Fig. S1A). The intersection between the cortex and thalamus revealed 14 shared molecules (*SLC1A2, LAMP5, IFI6, STAT1, CD68, SERPINA3, GPNMB, APOC1, TYROBP, SRGN, RGS1, SAT1, NPC2, BST2*) (Fig. 1B). Kyoto Encyclopedia of Genes and Genome (KEGG) pathway analysis revealed enrichment of immune and inflammatory signaling pathways exclusive to the ICH group. These differential pathways were detected within the cortex (inflammatory response, antigen processing and presentation, and proteins in the endoplasmic reticulum) and the thalamus (inflammatory response, phagosome activity and complement activation) (Fig. 1C). To distinguish between different cell types in the brain tissue, sections were stained for the neuronal marker NeuN, astrocyte marker GFAP, and microglia marker Iba1, and imaged on the GeoMx DSP (Supplementary Fig. S1B). Compared with the Control, neuroinflammatory response cells (GFAP^+^ astrocytes and Iba1^+^ microglia) increased significantly in the cortex and thalamus after ICH (Fig. 1D). Gene set enrichment analysis showed significant upregulation of genes related to inflammatory responses in both the cortex and thalamus (*IFI6, CD68, SERPINA3, TYROBP, BST2*). Among these, SERPINA3 was the most abundantly expressed upregulated gene (Fig. 1E). Further analysis revealed that SERPINA3 was the only upregulated gene in both cortical and thalamic GFAP^+^ astrocyte cells (Fig. 1F). Among the three segmentations, SERPINA3 expression increased in the GFAP^+^ segmentation after ICH (Fig. 1G). In situ immunostaining confirmed that SERPINA3 is predominantly expressed by astrocytes, with relatively low expression in microglia and neurons following ICH (Fig. 1H).

### Serpina3n was consistently elevated in the acute and chronic phases of ICH mouse models

**Fig. 2:**
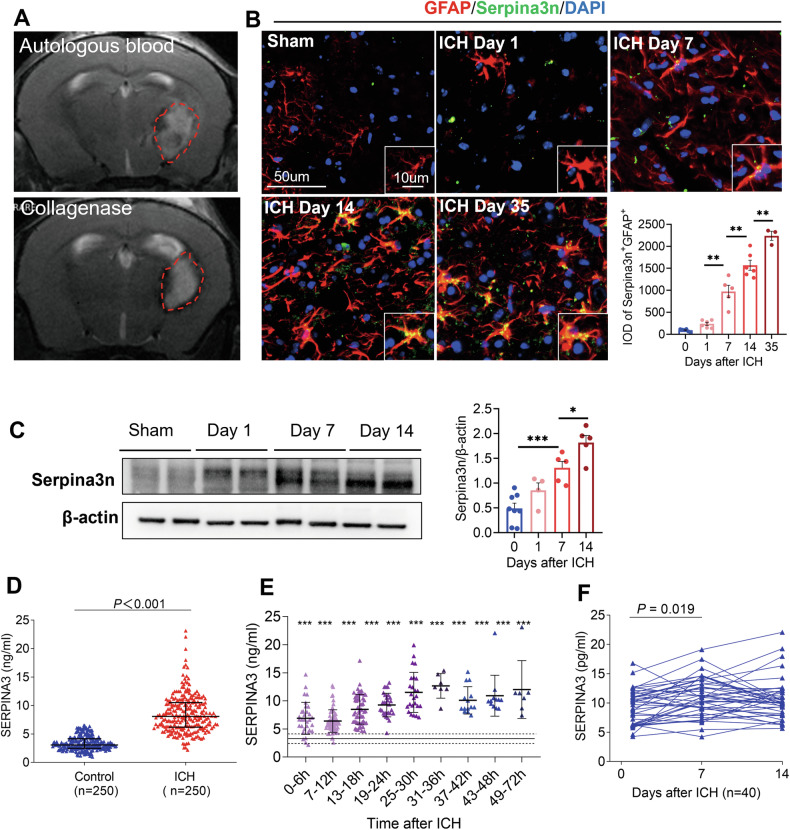
The expression of Serpina3n/SERPINA3 in ICH mouse brain and blood from patients with ICH. **A** The ICH model was induced by collagenase and autologous blood in C57BL/6 mice. **B** Immunostaining and quantification analysis of SerpinA3n+ GFAP+ in the thalamus at various time points after ICH induction in mice. *n* = 5 mice per group. Scale bar, 50 μm (Insert: 20 μm). One-way ANOVA. Error bars represent s.e.m. **C** Western blot analysis showing SerpinA3n protein levels at various time points after ICH induction in mice. Quantification of SerpinA3n in brain homogenates of ICH mice. *n* = 5 mice per group. One-way ANOVA, Error bars represent s.e.m. **D** Comparison of plasma SERPINA3 levels between ICH patients (within 72 hours of ictus) and healthy controls. Controls, *n* = 250; ICH patients, *n* = 250. Data are shown as the mean ± SEM. **E** Plasma SERPINA3 concentrations based on the time from ICH onset to blood collection. The median plasma SERPINA3 concentration in controls (3.06 ng/ml) is depicted as a solid horizontal line, and horizontal dotted lines denote the interquartile range (IQR: 2.48–4.15 ng/ml). *n* = 250 for controls. **F** Longitudinal analysis of plasma SERPINA3 levels in 40 ICH patients at day 1, day 7, and day 14 post-ictus. Continuous changes in plasma SERPINA3 levels were evaluated at the indicated time points. The statistical significance of the differences was calculated using the Mann–Whitney U test. *P < 0.05, **P < 0.01 and ***P < 0.001. *P* values are shown in the figure.

Having determined SERPINA3^+^ astrocytes as a prominent marker to chronic stage of ICH, we went on to investigate the changes of Serpina3n from PD1 to PD35, which includes both the acute and the long-term phases after ICH in two rodent models (Fig. 2A). Murine Serpina3n, the homolog of human SERPINA3, is a member of the serpin superfamily and is highly expressed in the brain, testis, lungs, thymus, and spleen. Compared to sham, Serpina3n and GFAP significantly increased at PD7 and became denser at PD35 in the thalamus, indicating that secondary thalamic neuroinflammation (Serpina3n^+^ GFAP^+^) began at PD7 and progressively developed through PD35. (Fig. 2B). In collagenase-induced ICH, mice similarly exhibited the changes of Serpina3n (Supplementary Fig. S2A). Increased Serpina3n protein levels were observed at the chronic phases of ICH compared with control mice (Fig. 2C and Supplementary Fig. S2B). Taken together, the rapid upregulation of Serpina3n in the acute phase, along with its sustained elevation in the chronic phase, suggests that Serpina3n may contribute to the regulation of chronic neuroinflammation following ICH.

### Plasma Serine proteinase inhibitor NA3 (SERPINA3) is elevated and progressively increases following ICH in patients

To explore the diagnostic and prognostic potential of SERPINA3 as a biomarker, we enrolled 250 ICH patients and 250 healthy controls. The demographic and clinical information of both groups was collected and is presented in Supplementary Table S1. The control group was matched to the ICH patients in terms of age and gender (Supplementary Table S1). Notably, plasma concentrations of SERPINA3 were significantly elevated in ICH patients compared with controls (3.07 ng/mL vs. 8.08 ng/mL, *P* < 0.001) (Fig. 2D). SERPINA3 levels were not associated with age or sex. (Supplementary Fig. S3A, B). We further classified patients based on the time from symptom onset to blood collection (0 to 12 hours, 12 to 36 hours, and 36 to 72 hours), allowing us to trace the changes in plasma SERPINA3 levels from disease onset to 3 days post-ictus. Plasma SERPINA3 levels peak at approximately 36 hours after admission and then declined to a relatively lower level until day 3 post-stroke (Fig. 2E). However, pairwise comparisons of SERPINA3 levels among the three time periods revealed no significant difference between the 12 to 36 hour and 36 to 72 hour intervals (*P* = 0.685; Supplementary table S2,S3). To further characterize the change in SERPINA3 levels from the acute to subacute phases, we measured plasma SERPINA3 in a subset of patients identified a subsequent increase on day 7 and 14 after ICH (Fig. 2F). These data suggest the dynamics of plasma SERPINA3 involve a gradual increase during the acute phase, followed by persistence after reaching a peak, consistent with the temporal characteristics of neuroinflammation.

### Plasma SERPINA3 correlates with the disease severity of intracerebral hemorrhage in the acute phase

To determine the relationship between SERPINA3 and acute brain tissue damage, we evaluated the correlation between plasma SERPINA3 levels and clinical measures at the time of blood collection. Hemorrhagic volume, neurological deficit, and coma severity were assessed using the ABC/2 formula, NIHSS and GCS, respectively. Analyses were performed for all patients with ICH and stratified by hemorrhage location, ventricular extension, and surgical intervention. In all patients with ICH, higher SERPINA3 levels were associated with worse outcomes in all three assessments. SERPINA3 was positively correlated with ABC/2 scores (*P* < 0.001; Table 1) and NIHSS scores (*P* < 0.001; Table 2) and negatively correlated with GCS scores (*P* < 0.001; Table 3) after adjusting for the time between stroke onset and blood collection, as well as other potential confounding factors (all *P* < 0.001). In stratified analyses, SERPINA3 levels were significantly associated with ABC/2, NIHSS, and GCS scores in patients with supratentorial hemorrhage, after adjusting for time from hemorrhage to blood collection, and other potential confounding variables. However, in patients with infratentorial hemorrhage, the correlation between SERPINA3 and ABC/2, NIHSS, GCS scores was no longer significant. We also assessed whether ventricular extension and surgical treatment affected SERPINA3 levels. SERPINA3 remained correlated with ABC/2, NIHSS, and GCS scores regardless of ventricular involvement or surgical intervention (Tables 1–3**)**. Similar findings were observed when stratifying patients by time from ICH to blood draw (Supplementary table S4–S6). Notably, SERPINA3 levels on day 7 were not associated with ABC/2, NIHSS and GCS scores at admission (Supplementary Table S7).

**Table 2:** Association of SERPINA3 concentrations with NIHSS scores at blood collection.

	Adjusting for the time from hemorrhage to blood collection			Adjusting for the time from hemorrhage to blood collection, age at blood collection, sex, current smoking, hypertension, cerebrovascular disease, and diabetes	
Variable types	*N*	β (95%CI)	*P*-value	β (95%CI)	*P*-value
Overall	250	1.34 (0.97, 1.71)	<0.001	1.21 (0.84, 1.58)	<0.001
**Hemorrhage location**					
No	220	1.3 (0.93, 1.67)	<0.001	1.22 (0.84, 1.60)	<0.001
Yes	30	1.77 (0.08, 3.45)	0.040	0.35 (−1.68, 2.38)	0.723
**Ventricular extension**					
No	182	1.24 (0.83, 1.65)	0.001	1.12 (0.70, 1.54)	<0.001
Yes	68	1.24 (0.83, 1.65)	0.001	1.12 (0.70, 1.54)	0.002
**Surgery**					
No	140	0.73 (0.22, 1.23)	0.005	0.6 (0.09, 1.11)	0.022
Yes	110	0.9 (0.36, 1.44)	0.001	0.88 (0.32, 1.44)	0.002

*β* regression coefficient; *CI* confidence interval. The *β* values, 95% CIs, and *p*-values were obtained from the linear regression models. *β* values were interpreted as the change in mean NIHSS scores for each fold of SERPINA3 concentration. *NIHSS* National Institutes of Health Stroke Scale.

**Table 3:** Association of SERPINA3 concentrations with GCS scores at blood collection.

	Adjusting for the time from hemorrhage to blood collection			Adjusting for the time from hemorrhage to blood collection, age at blood collection, sex, current smoking, hypertension, cerebrovascular disease, and diabetes	
Variable types	*N*	β (95%CI)	*P*-value	β (95%CI)	*P*-value
Overall	250	−0.37 (−0.48, −0.25)	<0.001	−0.34 (−0.46, −0.22)	<0.001
**Hemorrhage location**					
No	220	−0.35 (−0.47, −0.24)	<0.001	−0.34 (−0.46, −0.21)	0.001
Yes	30	−0.52 (−0.98, −0.06)	0.030	0.01 (−0.59, 0.61)	0.970
**Ventricular extension**					
No	182	−0.32 (−0.45, −0.20)	<0.001	−0.31 (−0.43, −0.18)	<0.001
Yes	68	−0.41 (−0.67, −0.16)	0.002	−0.36 (−0.62, −0.10)	0.007
**Surgery**					
No	140	−0.24 (−0.39, −0.08)	0.003	−0.20 (−0.35, −0.04)	0.016
Yes	110	−0.24 (−0.42, −0.06)	0.010	−0.23 (−0.42, −0.04)	0.016

*β* regression coefficient; *CI* confidence interval. The β values, 95% CIs, and p-values were obtained from the linear regression models. β values were interpreted as the change in mean GCS scores for each fold of SERPINA3 concentration. *GCS* Glasgow score.

### Plasma SERPINA3 correlates white matter injury and cognitive status at 6 months after ICH

**Fig. 3:**
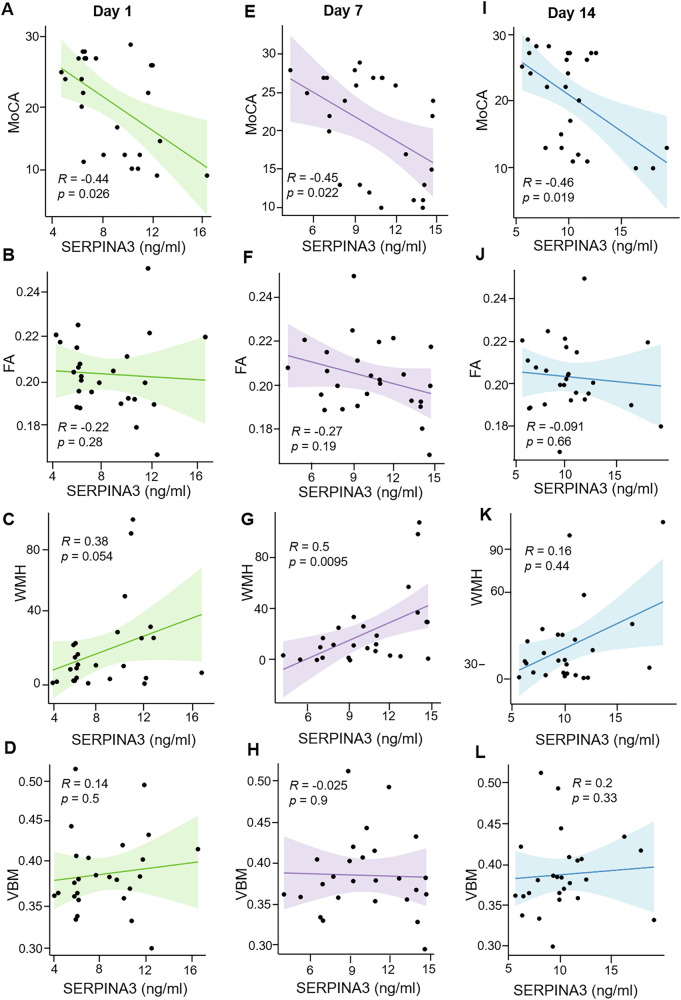
Correlation of plasma SERPINA3 at days 1, 7, and 14 with image markers and cognitive status at 6 months in ICH patients. Twenty-six patients underwent MRI and cognitive evaluation six months post-ICH. Plasma SERPINA3 concentrations were collected on days 1, 7, and 14 after ICH onset. The associations between plasma SERPINA3 concentrations and imaging markers, as well as cognitive status, were evaluated using Spearman’s test. **A**–**D** Correlation between plasma SERPINA3 levels on 1 day and fractional anisotropy (FA), voxel-based morphometry (VBM), white matter hyperintensities (WMH), and Montreal Cognitive Assessment (MoCA) scores. **E**–**H** Correlation between plasma SERPINA3 levels on day 7 and FA, WBM, WMH, and MoCA. **I**–**L** Correlation between plasma SERPINA3 levels on day 14 with FA, WBM, WMH, and MoCA. *ICH* intracerebral hemorrhage, *MoCA* Montreal Cognitive Assessment, *WMH* white matter hyperintensities, *VBM* voxel-based morphometry, *FA* fractional anisotropy. *n* = 26, Spearman’s rank correlation test was used, and *R* and *P* values are shown in the figure.

To determine the relationship between plasma SERPINA3 and chronic brain tissue damage following ICH, we conducted MRI scans on 26 patients 6 months post-ICH and evaluated their cognitive status using the Montreal Cognitive Assessment (MoCA). We analyzed the associations between plasma SERPINA3 levels within 72 hours, 7 days, and 14 days post-ICH, and MRI markers, including (i) white matter hyperintensities (WMH) volume, (ii) the structural integrity of white matter fibers evaluated by fractional anisotropy (FA), (iii) gray matter/white matter density or volume measured by voxel-based morphometry (VBM), and cognitive function. Plasma SERPINA3 at 7 days were positively correlated with WMH value (r = 0.5, *P* = 0.0095), while no significant correlation was observed for FA and VBM at any time points. Additionally, plasma SERPINA3 was negatively correlated with MoCA scores 6 months post-ICH, with the strongest correlation observed for SERPINA3 levels at day 14 (r = −0.46, *P* = 0.019). In previous research, we found that plasma neurofilament light chain (NfL) predicts long-term neurological outcomes in ICH patients [10]. Notably, plasma SERPINA3 levels were significantly correlated with plasma NfL levels (*r* = 0.21, *P* = 0.0009, Supplementary Fig. S3C). These findings indicate that plasma SERPINA3, particularly in the subacute phase, is closely associated with white matter hyperintensities and cognitive function six months after ICH (Fig. 3).

### Plasma SERPINA3 correlates with long-term outcomes of patients with intracerebral hemorrhage

During follow-up, 48 patients died, and the mRS of the remaining 202 patients was repeatedly assessed at 1, 3, 6, and 12 months post-ICH. This allowed us to examine the relationship between SERPINA3 and long-term outcomes in patients with ICH. Higher SERPINA3 levels within 72 hours were associated with an mRS ≥ 3 at 1, 3, 6, and 12 months, even after adjusting for the time between ICH onset and blood draw and other potential confounding variables (*P* < 0.001). We also examined whether the association between elevated SERPINA3 and worse mRS was independent of NIHSS and hemorrhage volume. After adjusting for NIHSS at the time of blood collection, the association between SERPINA3 and mRS at 1, 3, 6 or 12 months remained significant (*P* = 0.014 at 1 month, *P* = 0.001 at 3 months, *P* < 0.001 at 6, 12 months). Further adjustments for ABC/2 maintained the significance of the association between elevated SERPINA3 within 72 h and mRS at 3, 6 or 12 months (*P* = 0.009 at 3 months, *P* < 0.001 at 6, 12 months). The association remained significant even after adjusting for both NIHSS and ABC/2 (*P* = 0.02 at 3 months, *P* = 0.005 at 6 months, *P* < 0.001 at 12 months) (Table 4). These data indicate that the association between elevated SERPINA3 levels and an increased risk of mRS ≥ 3 at 3, 6, or 12 months after ICH is independent of NIHSS and ABC/2. Additionally, SERPINA3 levels on days 7 and 14 post-ICH were not significantly associated with mRS ≥ 3 (Supplementary table S8).

**Table 4:** Association of SERPINA3 within 72 hours with modified Rankin scale at different follow-up times in ICH patients.

	Adjustment for the time from stroke to blood collection		Adjustment for the time from stroke to blood collection, age, sex, current smoking, hypertension, cerebrovascular disease history, and diabetes		Adjustment for NIHSS at blood collection, age, sex, current smoking, hypertension, cerebrovascular disease history, and diabetes		Adjustment for the ABC/2, age, sex, current smoking, hypertension, cerebrovascular disease history, and diabetes		Adjustment for the NIHSS at blood collection, ABC/2, age, sex, current smoking, hypertension, cerebrovascular disease history, and diabetes	
Follow-up time	OR (95%CI)	*P* value	OR (95%CI)	*P* value	OR (95%CI)	*P* value	OR (95%CI)	*P* value	OR (95%CI)	*P* value
1 month	1.32 (1.14, 1.53)	<0.001	1.28 (1.10, 1.49)	0.002	1.24 (1.04, 1.47)	0.014	1.18 (1.00, 1.38)	0.051	1.18 (0.98, 1.43)	0.084
3 months	1.34 (1.18, 1.52)	<0.001	1.32 (1.15, 1.50)	<0.001	1.29 (1.11, 1.50)	0.001	1.21 (1.05, 1.40)	0.009	1.22 (1.03, 1.43)	0.020
6 months	1.46 (1.28, 1.66)	<0.001	1.43 (1.25, 1.64)	<0.001	1.43 (1.23, 1.66)	<0.001	1.46 (1.25, 1.70)	<0.001	1.41 (1.18, 1.68)	0.005
12 months	1.55 (1.35, 1.78)	<0.001	1.53 (1.32, 1.76)	<0.001	1.51 (1.29, 1.77)	<0.001	1.54 (1.28, 1.85)	<0.001	1.55 (1.27, 1.89)	<0.001

*CI* confidence interval. Odds ratios, 95% CIs, and *p*-values resulting from binary logistic regression models. ORs were interpreted as multiplicative increases in the odds of a modified Rankin Scale score ≥ 3 for each fold increase in SERPINA3 concentrations. The complete multivariable analysis was adjusted for the time from stroke to blood collection, age, sex, current smoking, hypertension, cerebrovascular disease, and diabetes. The bleeding volume of intravascular hemolysis (IVH) was calculated using a 3D slicer. *mRS* modified Rankin Scale, *NIHSS* National Institutes of Health Stroke Scale, *GCS* Glasgow Coma Score.

We also examined the ability of SERPINA3 to independently predict an mRS ≥ 3 by estimating the AUC with and without SERPINA3 included in the multivariable logistic regression model. An AUC of 1.0 indicates perfect predictive ability, while an AUC equal to 0.5 represents predictive ability equal to chance. Including SERPINA3 in the model increased the AUC from 0.679 to 0.901 (when considering NIHSS) or from 0.866 to 0.926 (when considering ABC/2) at 3 months; from 0.679 to 0.887 (NIHSS) or from 0.853 to 0.910 (ABC/2) at 6 months; and from 0.687 to 0.868 (NIHSS) or from 0.840 to 0.891 (ABC/2) at 12 months (Supplementary table S9).

### Plasma SERPINA3 predicts the survival of patients with intracerebral hemorrhage

**Fig. 4:**
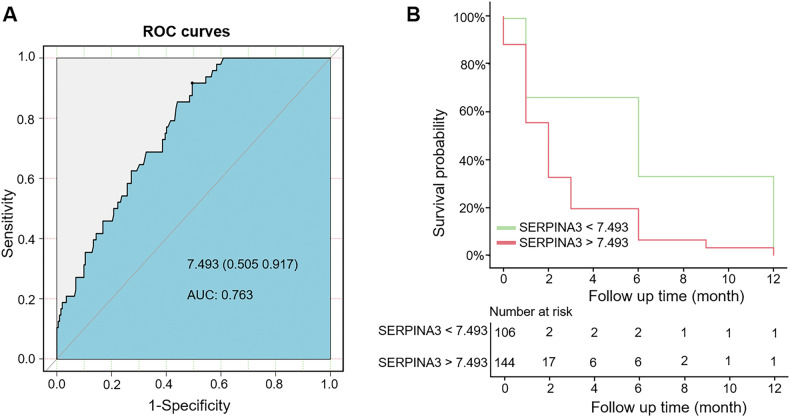
SERPINA3 concentrations measured within 72 hours of disease onset and survival within the 12-month interval. The association between SERPINA3 concentrations measured within 72 hours of disease onset and12-month survival was evaluated using Cox proportional hazard regression models. **A** Receiver operating characteristic (ROC) curve assessing the diagnostic accuracy of SERPINA3 in predicting survival versus mortality outcomes. The AUC, and criterion value are shown. AUC = 0.763. **B** Kaplan–Meier curve with a log-rank test of the predictor above and below the cut-off value. (SERPINA3 = 7.493 ng/ml).

We next investigated whether SERPINA3 could predict survival in patients with ICH. The date of blood collection was set as the baseline for survival analysis, and patients were censored at the date of their last follow-up or death. During the follow-up period, 48 patients (19.2%) died. Higher SERPINA3 was significantly associated with poorer survival in all patients with ICH [1.0017, odds ratio (OR) (95% CI), 1.0004, 1.0031; *P* = 0.01], after adjusting for the time from ICH onset to blood collection. (Supplementary table S10). The AUC was calculated to assess the ability of SERPINA3 to independently predict survival at 12 months post-ICH. SERPINA3 levels differentiated survivors from non-survivors with an AUC of 0.763 at 12 months. The optimal cut-off point for SERPINA3 was determined to be 7.493 ng/ml. Further analysis using the Kaplan-Meier survival curve and log-rank test of the predictor revealed that a plasma SERPINA3 level above 7.493 ng/ml at admission was associated with a significantly increased risk of death (*P* < 0.001, Fig. 4).

## Discussion

SERPINA3 is a secreted peptidase inhibitor from the Serpin family, whose expression is induced by inflammation and nerve injury [20]. Previous studies have shown that central glial cells can induce SERPINA3 over-expression, making it a potential marker of apoptosis and hypothalamic inflammation during central nervous system injury [21–23]. Elevated SERPINA3 levels have been found in cerebrospinal fluid of patients with Alzheimer’s disease, correlating with disease severity [24]. SERPINA3 may signify disease status and progression of multiple sclerosis (MS) [25]. Additionally, astrocyte-derived SERPINA3 promotes neuroinflammation and epileptic seizures [26]. In ischemic stroke, SERPINA3 has been proposed as a potential replacement of glial fibrillary acidic protein (GFAP), a traditional biomarker for activated astrocytes [27]. Our study’s findings regarding the association between plasma SERPINA3 levels and clinical outcomes in ICH patients align with a prior report involving a smaller cohort of ICH patients (*n* = 40) [28]. However, based on the combination of spatial transcriptomic and in situ staining of ICH patients’ brain autopsy tissue, as well as models, our study offers several new insights. These include the consolidation of the presence of SERPINA3 and its related pathways; correlation between plasma SERPINA3 and long-term outcomes up to 12 months post-ICH; the predictive value of SERPINA3 for patient survival; the dynamic changes in plasma SERPINA3 from acute to subacute stages of ICH and its association with brain structural changes on MRI during the chronic phase. These findings support future studies to explore changes in plasma SERPINA3 in the chronic stage and its potential role in late-onset comorbidities, such as cognitive decline, among ICH survivors.

In the acute stage of cerebral hemorrhage, the inflammatory-immune response is activated, with commonly observed acute inflammation markers including leukocytes, neutrophils, lymphocytes, and monocytes in the peripheral blood [15, 29]. However, there is currently no evidence that these markers can predict long-term chronic nerve damage in months or years after ICH. Our study identified a persistent inflammatory response in the chronic phase of ICH, shedding light on the dynamic evolution of these indicators over time and their corresponding clinical correlations. Plasma SERPINA3 levels peak at approximately 36 hours after admission and then declined to a relatively lower level until day 3 post-stroke. However, the continued detection of SERPINA3 in a subset of patients identified a subsequent increase on day 7 and 14 after ICH. Meanwhile, intracerebral SERPINA3 in astrocytes gradually increases as the ICH progresses. One possible explanation is that inflammation and innate immune cells activation are present throughout the entire course of intracerebral hemorrhage [12]. Thus, plasma SERPINA3 helped sensitively trace inflammation post-ICH, suggesting that the inflammatory response is already present in the early acute phase and continues throughout the course of the injury. Furthermore, we found that plasma SERPINA3 levels within 72 hours were closely associated with hemorrhage volume and clinical manifestations at admission. SERPINA3 levels measured 7 days post-ictus were correlated with white matter hyperintensities and poor cognitive performance at 6 months. These findings suggest that stroke-inflammation markers, such as SERPINA3, may have different clinical significance depending on the stage of cerebral hemorrhage. Before SERPINA3 can be incorporated as a clinical biomarker, additional research is needed to ensure its accuracy and reliability in clinical practice.

At present, there is substantial evidence suggesting that NfL, S100β, and GFAP, as markers of axonal injury and astrocytes, can predict the disease prognosis [30–33]. Our previous research has shown that plasma NfL is reliably associated with long-term outcomes and survival in patients with ICH [10]. Plasma GFAP appears to be a sensitive and specific biomarker for distinguishing ICH from both acute ischemic stroke (AIS) and other acute neurological disorders [34]. Interestingly, this study found that plasma SERPINA3 levels significantly correlated with plasma NfL levels. Unlike these markers, which directly reflect nerve cell damage, SERPINA3 primarily targets multiple types of immune cells and inhibits the release of serine proteases to maintain immune balance. It acts as a marker of inflammation and astrocyte activation [35–37]. The findings of this study provide strong evidence for the role of SERPINA3 in cerebral hemorrhage and its potential use as a prognostic biomarker. However, combining SERPINA3 with other potential markers may offer a more comprehensive strategy for future clinical practice, given the complexity of chronic cerebral hemorrhage’s pathophysiological characteristics and the diversity of long-term prognoses.

Several limitations of this study should be noted. First, although spatial transcriptomic results indicate increased expression of SERPINA3 in the brain during chronic intracerebral hemorrhage, the exact mechanisms of SERPINA3’s effects on neural function following ICH remain unknown. Second, due to the nature of the study, most patients could not participate in on-site follow-up assessments or have repeated measurements of SERPINA3 levels at a later stage. Additionally, MRI measurements were only available for a small subset of the study population, limiting our ability to investigate specific associations and late-onset changes in patients with cerebral hemorrhage. Despite these limitations, our findings provide strong evidence for the potential value of plasma SERPINA3 as a biomarker for predicting the long-term prognosis of spontaneous intracerebral hemorrhage. The integration of spatial transcriptomic sequencing, pathological staining, and serological analysis offers comprehensive evidence supporting the involvement of SERPINA3 in the pathogenesis of cerebral hemorrhage and its prognostic significance in clinical practice. Further research is necessary to fully elucidate the diagnostic and therapeutic implications of SERPINA3 in cerebral hemorrhage management.

## Supplementary information

Supplementary material

Western Blot Result

## Data Availability

All raw RNA-seq data (fastq files) were deposited to GSA (https://ngdc.cncb.ac.cn/gsa/) under accession number HRA009250.
