# Supplementary material for: SERPINA3 predicts long-term neurological outcomes and mortality in patients with intracerebral hemorrhage
Source: Cell Death Dis. 2025 Mar 29;16(1):218. doi: 10.1038/s41419-025-07551-x (PMC11954896; doi:10.1038/s41419-025-07551-x)
Supplement: Supplementary file 1 — Supplementary material [file 41419_2025_7551_MOESM1_ESM.docx]

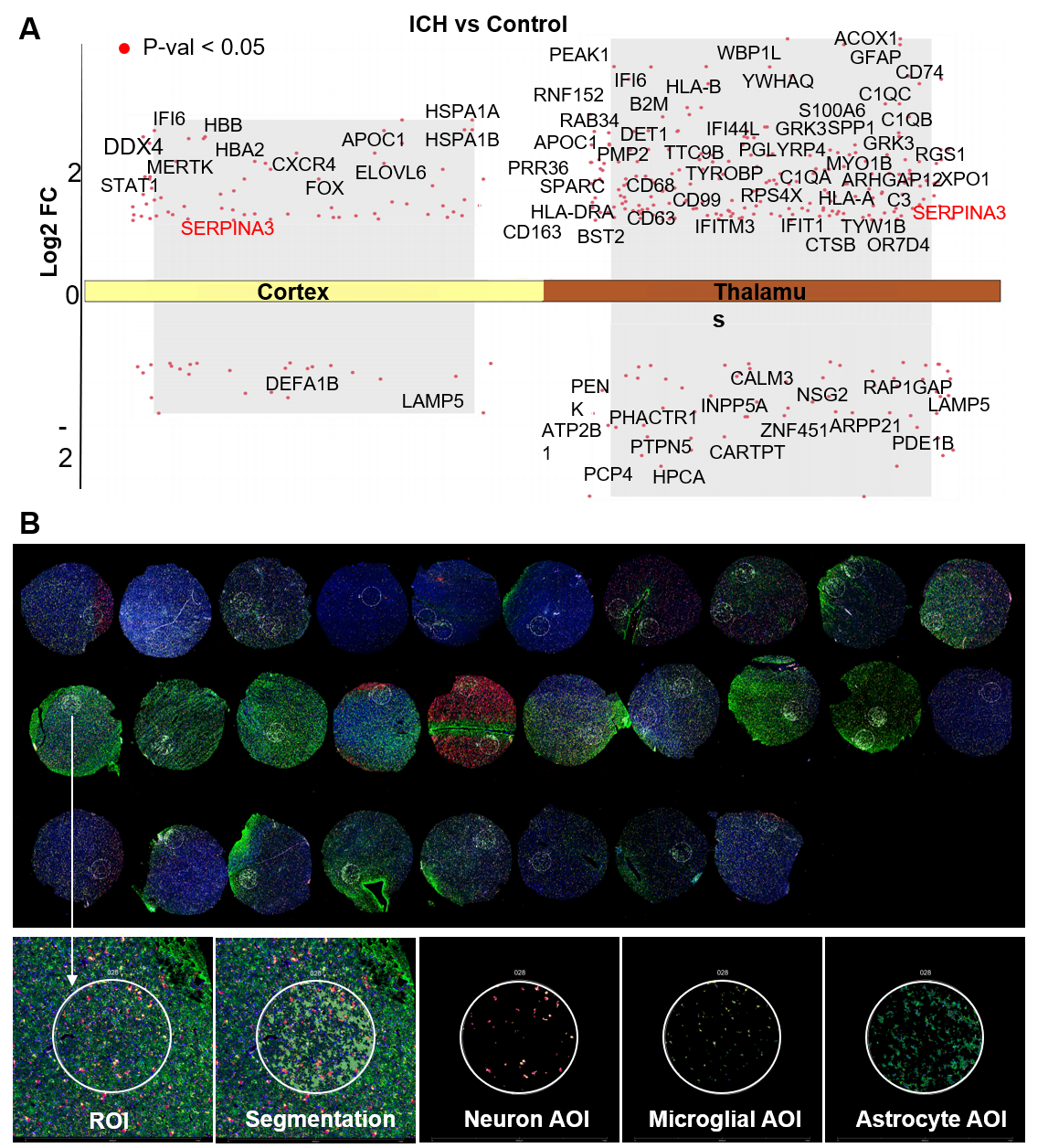


**Fig. S1**. (A) Volcano plot comparing differentially expressed genes in the thalamus and cortex of ICH patients compared to controls. Y-axis represents average log2 FC. Red indicates an adjusted P value < 0.05. The gene shown in the figure is the log2 (Fold change) cutoffs (|FC| >1.5). (B) Up: Representative image of TMA acquired using the GeoMx DSP system. The numerical annotations corresponding to the respective ROI numbers. Down: Representative ROI (ROI no. 28) and AOIs annotated based on histology by pathologists and immunofluorescence staining with the morphological markers and the compartmentalized image created by fluorescence colocalization. One 300 μM ROI per core was selected for DSP. GFAP (green), NeuN (red), Iba-1 (yellow). The scale bar is 500 μm.


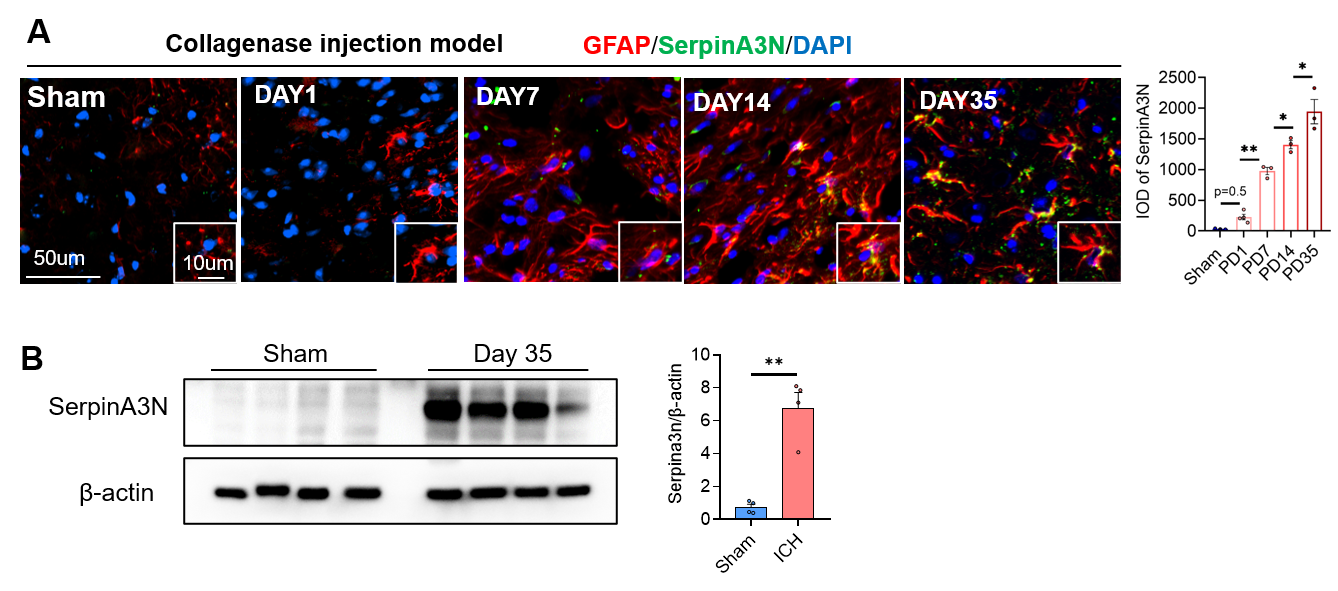


**Fig. S2.** (A) Immunostaining and quantification analysis of SerpinA3n+ GFAP+ in the thalamus at various time points after collagenase injection model. n = 5 for each group, Scale bar, 50 μm (Insert: 20 μm). (B) Western blot analysis showing SerpinA3n protein levels at day 35 after ICH induction in mice. Quantification of SerpinA3n in brain homogenates of ICH mice. n = 4 mice per group. One-way ANOVA, Error bars represent s.e.m.


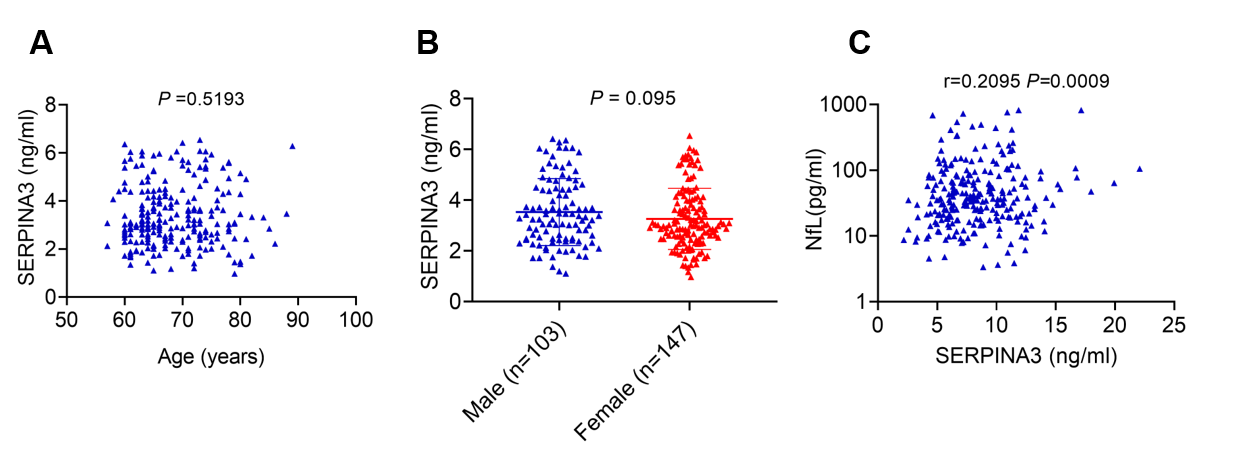


**Fig. S3. Blood** **SERPINA3 concentrations in healthy controls correlated with age and sex respectively.**

(A) Linear regression plot displaying the correlation between age and plasma SERPINA3 levels in healthy individuals (Spearman’s r=0.04, P = 0.519, N=250). (B) Interleaved scatter graph shows plasma SERPINA3 values for the males and females (P = 0.095). (C) Correlation plots between SERPINA3 and NfL levels in the plasma of patients with ICH (Spearman’s r=0.2095, P = 0.0009, N=250). NfL = neurofilament light chain; r = correlation coefficient.

| **Table S1. Characteristics of controls and ICH patients.** | | | | | |
| --- | --- | --- | --- | --- | --- |
| **Variable**, **No. (%)** | **Controls**  **(n=250)** | **All ICH patients**  **(n=250)** | | **Patients with blood samples available at days 7 and 14 (n=40)** | |
| Age, years (median (minimum, maximum)) | 67 (57, 89) | 62 (27, 84) | | 63 (31, 77) | |
| Male | 103 (41.2) | 135 (54.0) | | 24 (60.0) | |
| **Medical history** |  |  | |  | |
| Diabetes, | 61 (24.4) | 26 (10.4) | | 4 (10.0) | |
| Hypertension, | 116 (46.4) | 155 (62.0) | | 24 (60.0) | |
| Cerebrovascular Disease | 3 (1.2) | 39 (15.6) | | 5 (12.5) | |
| Current smoking status | 67 (26.8) | 100 (40.0) | | 17 (42.5) | |
| **Clinical evaluations at admission** | | | | | |
| Glasgow Coma Scale |  |  | |  | |
| 15–14 | N/A | 100 (40.0) | | 15 (37.5) | |
| 13–9 | N/A | 112 (44.8) | | 21 (52.5) | |
| 8–3 | N/A | 38 (15.2) | | 4 (10.0) | |
| NIHSS |  |  | |  | |
| 0–4 | N/A | 15 (6.0) | | 2 (5.0) | |
| 5–15 | N/A | 113 (45.2) | | 15 (37.5) | |
| 16–20 | N/A | 54 (21.6) | | 11 (27.5) | |
| 21–43 | N/A | 68 (27.2) | | 12（30） | |
| **Hemorrhagic features on CT** | | | | | |
| Location of hemorrhage |  |  | |  | |
| Spontaneous supratentorial hemorrhage | N/A | 30 (12.0) | | 7 (17.5) | |
| Infratentorial supratentorial hemorrhage | N/A | 220 (88.0) | | 33 (82.5) | |
| Hemorrhage volume, Median (IQR), mL | N/A | 16 (32) | | 20 (40) | |
| Intraventricular bleeding | N/A | 68 (27.2) | | 16 (40.0) | |
| **Surgical treatment** | N/A | 110 (44. 0) | | 20 (50.0) | |
| **Outcomes** |  |  | |  | |
| Death | N/A | 48 (19.2) | | 4 (10.0) | |
| Modified Rankin Scale score | N/A | mRS < 3 mRS≥3 | | mRS < 3 mRS≥3 | |
| 1 month | N/A | 43 (17.2) 176 (70.4) | | 5 (12.5) 32 (80.0) | |
| 3 months | N/A | 75 (30.0) 135 (54.0) | | 11 (27.5) 25 (62.5) | |
| 6 months | N/A | 107 (42.8) | 98 (39.2) | 19 (47.5) | 17 (42.5) |
| 12 months | N/A | 127 (50.8) | 85 (34.0) | 21 (52.5) | 15 (37.5) |

Data are presented as numbers (%) or as indicated. NIHSS, National Institutes of Health Stroke Scale; IQR, interquartile range; N/A, not available.

| **Table S2. Comparisons of plasma SERPINA3 concentrations between controls and ICH patients.** | | | | | | |
| --- | --- | --- | --- | --- | --- | --- |
|  |  |  | **Unadjusted** | | **Adjusting for age at blood draw and sex** | |
| **Disease group** | N | Median (minimum, maximum)  SERPINA3 | β (95%CI) | *P*-value | β (95%CI) | *P*-value |
| Controls | 250 | 3.07 (0.98, 6.93) | 0.00 (reference) | N/A | 0.00 (reference) | N/A |
| ICH study patients | 250 | 8.08 (2.18, 23.13) | 5.28 (4.73, 5.83) | <0.001 | 4.88 (4.19, 5.57) | <0.001 |
| 0-12 hours | 99 | 6.29 (2.18, 14.70) | 3.30 (2.85, 3.74) | <0.001 | 2.98 (2.39, 3.58) | <0.001 |
| 12-36 hours | 118 | 9.38 (4.29, 19.92) | 6.31 (5.77, 5.75) | <0.001 | 5.87 (5.14, 6.60) | <0.001 |
| 36-72 hours | 33 | 10.06 (7.44, 23.13) | 7.54 (6.83, 8.24) | <0.001 | 8.16 (7.12, 9.22) | <0.001 |

β=regression coefficient; CI=confidence interval. β values, 95% CIs, and p-values result from linear regression models. β values are interpreted as the difference in the mean SERPINA3 concentration for the ICH group in comparison to controls. P-value < 0.001 is considered statistically significant.

| **Table S3. Comparison of SERPINA3 concentrations between the 0- to 12-hour, 12- to 36-hour, and 36- to 72-hour time periods in the ICH group.** | | |
| --- | --- | --- |
| **ICH group** | **N** | **Median (minimum, maximum)** |
| 0-12 hours | 99 | 6.29 (2.18, 14.7) |
| 12-36 hours | 99 | 9.50 (4.29, 19.90) |
| P-value for difference |  | <0.001 |
| 0-12 hours | 33 | 5.52 (2.58, 12.5) |
| 36-72 hours | 33 | 10.1 (7.44, 23.1) |
| P-value for difference |  | <0.001 |
| 12-36 hours | 33 | 9.00 (4.84, 19.9) |
| 36-72 hours | 33 | 10.00 (7.44, 22.1) |
| P-value for difference |  | 0.685 |
| P-values result from a paired Wilcoxon signed rank test. P-values <0.001 are considered statistically significant after correction for multiple testing. | | |

| **Table S4: Association of SERPINA3 concentrations with ABC/2 when stratifying patients by time from ICH to blood draw.** | | | | | |
| --- | --- | --- | --- | --- | --- |
|  | **N** | **Unadjusted** **analysis** | | **Multivariable analysis*** | |
| **Group** |  | **β (95%Cl)** | ***P*** | **β (95%Cl)** | ***P*** |
| 0-12 hours from ICH to blood draw (h) | 99 | 5.81 (3.76, 7.87) | <0.001 | 5.29 (3.04, 7.53) | <0.001 |
| 12-36 hours from ICH to blood draw (h) | 217 | 3.71 (2.22, 5.20) | <0.001 | 3.70 (2.24, 5.16) | <0.001 |
| 36-72 hours from ICH to blood draw (h) | 33 | 5.41 (3.68, 7.14) | <0.001 | 5.44 (3.55, 7.34) | <0.001 |
| β=regression coefficient; CI=confidence interval. β values, 95% Cl, and *P* values result from linear regression models. P-value < 0.001 is considered statistically significant. * Adjusting for the time from hemorrhage to blood collection, age at blood collection, sex, current smoking, hypertension, cerebrovascular disease, and diabetes. | | | | | |

| **Table S5: Association of SERPINA3 concentrations with NIHSS scores when stratifying patients by time from ICH to blood draw.** | | | | | |
| --- | --- | --- | --- | --- | --- |
|  | **N** | **Unadjusted** **analysis** | | **Multivariable analysis*** | |
| **Group** |  | **β (95%Cl)** | ***P*** | **β (95%Cl)** | ***P*** |
| 0-12 hours from ICH to blood draw (h) | 99 | 3.01 (2.22, 3.81) | <0.001 | 2.62 (1.77, 3.47) | <0.001 |
| 12-36 hours from ICH to blood draw (h) | 217 | 1.55 (1.14, 1.95) | <0.001 | 1.40 (0.99, 1.82) | <0.001 |
| 36-72 hours from ICH to blood draw (h) | 33 | 1.48 (0.49, 2.47) | 0.005 | 1.56 (0.54, 2.58) | 0.004 |
| β=regression coefficient; CI=confidence interval. β values, 95% Cl, and *P* values result from linear regression models. P-value < 0.001 is considered statistically significant. * Adjusting for the time from hemorrhage to blood collection, age at blood collection, sex, current smoking, hypertension, cerebrovascular disease, and diabetes. | | | | | |

| **Table S6: Association of SERPINA3 concentrations with GCS scores when stratifying patients by time from ICH to blood draw.** | | | | | |
| --- | --- | --- | --- | --- | --- |
|  | **N** | **Unadjusted** **analysis** | | **Multivariable analysis*** | |
| **Group** |  | **β (95%Cl)** | ***P*** | **β (95%Cl)** | ***P*** |
| 0-12 hours from ICH to blood draw (h) | 99 | -0.84 (-1.11, -0.58) | <0.001 | -0.79 (-1.07, -0.50) | <0.001 |
| 12-36 hours from ICH to blood draw (h) | 217 | -0.41 (-0.55, -0.28) | <0.001 | -0.38 (-0.51, -0.24) | <0.001 |
| 36-72 hours from ICH to blood draw (h) | 33 | -0.53 (-0.73, -0.32) | <0.001 | -0.51 (-0.72, -0.29) | <0.001 |
| β=regression coefficient; CI=confidence interval. β values, 95% Cl, and *P* values result from linear regression models. P-value < 0.001 is considered statistically significant. * Adjusting for the time from hemorrhage to blood collection, age at blood collection, sex, current smoking, hypertension, cerebrovascular disease, and diabetes. | | | | | |

| **Table S7: Associations of SERPINA3 concentrations at day 7 and day 14 with ABC/2, NIHSS and GCS scores at blood draw.** | | | | | | |
| --- | --- | --- | --- | --- | --- | --- |
|  | **SERPINA3 concentrations day 7** | | | **SERPINA3 concentrations day 14** | | |
| **Variable types** | **N** | **β (95%Cl)** | ***P*** | **N** | **β (95%Cl)** | ***P*** |
| NIHSS | 40 | 0.29 (-0.71, 1.29) | 0.557 | 40 | 0.81 (0.01, 1.61) | 0.047 |
| ABC/2 | 40 | 1.77 (-1.06, 4.61) | 0.211 | 40 | 3.86 (1.85, 5.87) | <0.001 |
| GCS | 40 | -0.21 (-0.52, 0.10) | 0.170 | 40 | -0.30 (-0.54, -0.05) | 0.019 |
| β=regression coefficient; CI=confidence interval. β values, 95% Cl, and *P* values result from linear regression models. P-value < 0.001 is considered statistically significant. A rigorous multivariable analysis was not performed due to small sample size. NIHSS= National Institutes of Health Stroke Scale. GCS=Glasgow coma scale. | | | | | | |

| **Table S8: Association of SERPINA3 concentrations at day 7 and day 14 with mRS ≥ 3 scores at different follow-up times in ICH patients.** | | | | | | | | | | | | | | | |
| --- | --- | --- | --- | --- | --- | --- | --- | --- | --- | --- | --- | --- | --- | --- | --- |
|  | | | | **SERPINA3 concentrations day 7** | | | | | | **SERPINA3 concentrations day 14** | | | | | |
| Follow-up time | | | | **N** | **β (95%Cl)** | | | ***P*** | | **N** | **β (95%Cl)** | | | ***P*** | |
| 1 month | | | | 247 | 1.1(0.94,1.29) | | | 0.233 | | 247 | 1.16(1.01,1.32) | | | 0.033 | |
| 3 months | | | | 246 | 1.11(0.93,1.32) | | | 0.240 | | 246 | 1.17(1.02,1.36) | | | 0.029 | |
| 6 months | | | | 246 | 1.14(0.95,1.38) | | | 0.162 | | 246 | 1.24(1.07,1.43) | | | 0.006 | |
| 12 months | | | | 246 | 1.14(0.94,1.37) | | | 0.173 | | 246 | 1.23(1.07,1.43) | | | 0.007 | |
| β=regression coefficient; CI=confidence interval. β values, 95% Cl, and *P* values result from linear regression models. P-value < 0.001 is considered statistically significant. A rigorous multivariable analysis was not performed due to small sample size. mRS=modified Rankin Scale. | | | | | | | | | | | | | | | |
| **Table S9. Examination of the ability of SERPINA3 concentrations to independently predict 3, 6 and 12-month poorer mRS scores.** | | | | | | | | | | | | | | | |
|  | |  | | **Full multivariable model with NIHSS at blood collection** | | | | **Full multivariable model with ABC/2** | | | | | **Full multivariable model with ABC/2+NIHSS at blood collection** | | |
| **Follow-up time** | | Predictive ability measure | Without SERPINA3 | | | | With SERPINA3 | Without SERPINA3 | | With SERPINA3 | | | Without SERPINA3 | With SERPINA3 | |
| 3 months | | AUC | 0.679 | | | | 0.901 | 0.866 | | 0.926 | | | 0.947 | 0.966 | |
| 6 months | | AUC | 0.679 | | | | 0.887 | 0.853 | | 0.910 | | | 0.932 | 0.947 | |
| 12 months | | AUC | 0.687 | | | | 0.868 | 0.840 | | 0.891 | | | 0.932 | 0.945 | |
| AUC=area under the ROC curve. AUCs result from binary logistic regression models. This full multivariable was adjusted for time from ICH to blood draw, age at blood draw, sex, current smoking, hypertension, cerebrovascular disease, diabetes. | | | | | | | | | | | | | | | |

| **Table S10: Associations between SERPINA3 concentrations within 72 hours and survival after ICH.** | | |
| --- | --- | --- |
| **Variables** | **HR (95% CI)** | **P value** |
| mortality | 1.0017 (1.0004, 1.0031) | 0.01 |

Hazard ratios result from Cox proportional hazards regression models.
