# Supplementary figures and images for: SERPINA3 predicts long-term neurological outcomes and mortality in patients with intracerebral hemorrhage
Source: Cell Death Dis. 2025 Mar 29;16(1):218. doi: 10.1038/s41419-025-07551-x (PMC11954896; doi:10.1038/s41419-025-07551-x)

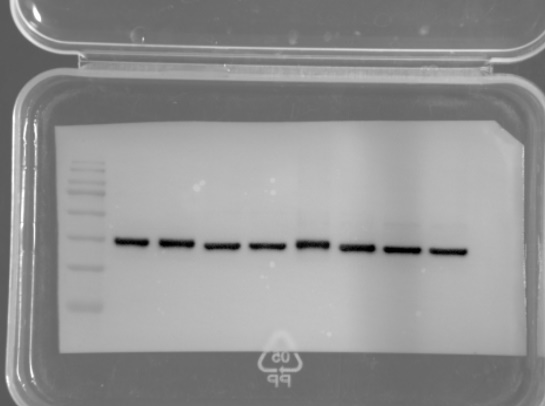

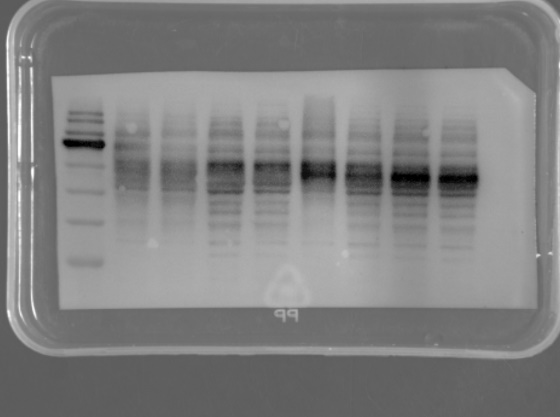

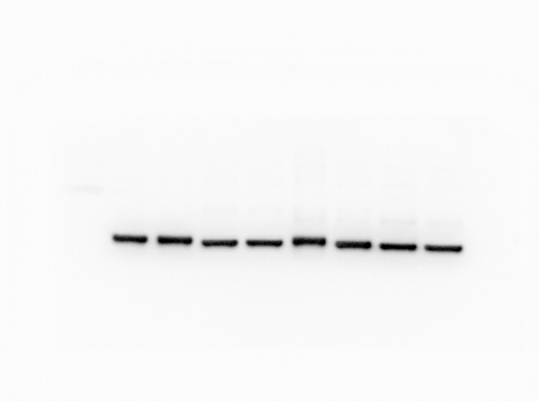

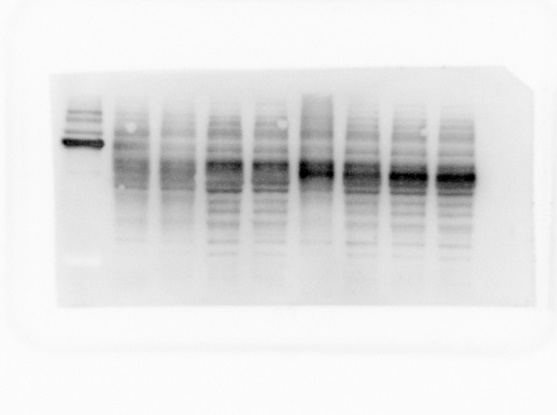

Supplement: Supplementary file 2 — Western Blot Result [file 41419_2025_7551_MOESM2_ESM.docx]
